# Shaver Suction Technique for Troubleshooting in Arthroscopic Double Foreign Body Removal From the Hip Joint

**DOI:** 10.7759/cureus.10911

**Published:** 2020-10-12

**Authors:** Aydin Budeyri, Mehmet C Cankus

**Affiliations:** 1 Department of Orthopaedics and Traumatology, Sanko University, Faculty of Medicine, Gaziantep, TUR; 2 The Shoulder Center, Baylor University Medical Center, Dallas, USA

**Keywords:** hip arthroscopy, suction stabilization technique, loose body removal

## Abstract

Most arthroscopic techniques provide easy invasive access and subsequent inspection of the lateral segments of the hip joint. However, it is a challenge to visualize medial segments of the hip joint using arthroscopic techniques. Hip arthroscopy offers minimally invasive access to the hip joint as compared to the standard open arthrotomy procedure. Yet, visualization of both the femoral head and acetabulum is difficult. The use of arthroscopic techniques in the diagnosis and treatment of hip-related disorders is still evolving, including great benefits for the postoperative healing and complications. The author describes a case of removing two loose bodies stuck in the superior basicervical rim of the femoral head of a 53-year-old man. The use of the inferomedial arthroscopic technique proved advantageous in preventing the shortcomings associated with standard arthrotomy and other arthroscopic mechanisms. Such shortcomings include the need for traction, alternate portals, and damage to the acetabular labrum and articular cartilage. Through this case report, the author establishes the effective use of hip arthroscopy in the removal of two loose bodies from the hip joint.

## Introduction

In 1931, Burman published his findings on hip arthroscopy. For the past 75 years, hip joint arthroscopy, including inspection and visualization of the hip joint, has been practiced as described by Burman [1]. The first arthroscopic removal of foreign bodies, such as a bullet from the hip joint of patients, was performed in 1998 [2]. According to Gross [3], the approach is widely used today in adults and children, for example, in shoulder and knee joints. However, the bony architecture of the hip joint makes the operative hip arthroscopic approach technically frustrating. The difficulty arises from the reduced accessibility and high risk of vascular and neurological complications. Despite the aforementioned challenges, hip arthroscopy remains an option for removing foreign bodies. With advanced arthroscopic techniques and equipment, surgeons can diagnose and treat disorders that previously required an open arthrotomy.

The hip arthroscopy technique is advantageous because it shortens the rehabilitation time and reduces subsequent discomfort. In addition, it allows exceptional access to the articular part of the femoral head. Another advantage of hip arthroscopy is the minimization of the disruption of soft tissue, thereby reducing the risk of post-operative stifling and avascular necrosis. Last but not least, capsuloligamentous structures, including the labrum remain undamaged, thus reducing probable postoperative instability.

The aim of this report is to describe a rare inferomedial technique that provides a more efficient approach in removing two loose bodies from a hip joint. The pearls and pitfalls regarding the intraarticular double foreign body removal have been highlighted. Furthermore, the report provides a troubleshooting technique to rely upon when an arthroscopic grasper’s claw gets broken and, even worse, becomes loose inside the hip joint.

## Case presentation

**Figure 1:**
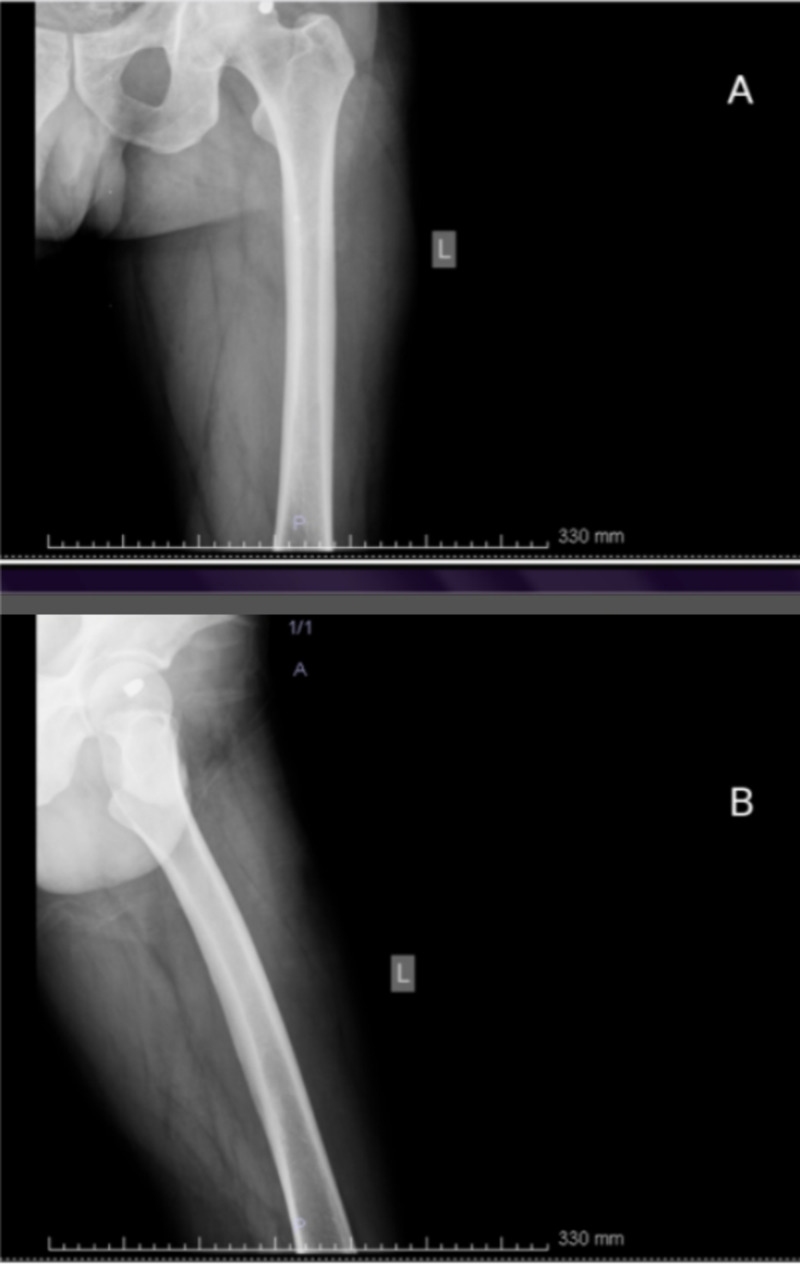
Computed tomographic scans A. Preoperative anteroposterior hip view, B. Preoperative lateral hip view

A 53-year-old, class I obese, male patient was presented to the emergency department. He had sustained a gunshot with a .765 caliber bullet injury on his left hip. Based on both physical and radiographic examination, no neurovascular injury was diagnosed. Computed tomographic scans identified the bullet and revealed that it was lodged inside the superior basicervical rim of the femoral head (Figures 1A-1B). Arthroscopic bullet removal from the hip joint was scheduled, and the patient’s informed consent was obtained.

The patient was taken into the operating room according to schedule, and preoperative anesthesia was administered. Then, routine anterolateral and anterior arthroscopic hip portals were established based on Guanche’s technique [4]. The arthroscopic shaver, spatula, probe, and grasper were introduced via the anterior portal. On the other hand, the arthroscopic scope was introduced via the anterolateral portal. Hip arthroscopy cannulas of 8.25 mm by 11 cm were introduced into the portals, respectively. During the operation, the bullet was embedded in the superior basicervical rim of the femoral head. An attempt was made to dislodge the bullet from the bone using a claw-sized arthroscopic grasper. The bullet was lodged tightly into the femoral head, which complicated the operation. An arthroscopic spatula was then used to raise the bullet off the bone with no success.

**Figure 2:**
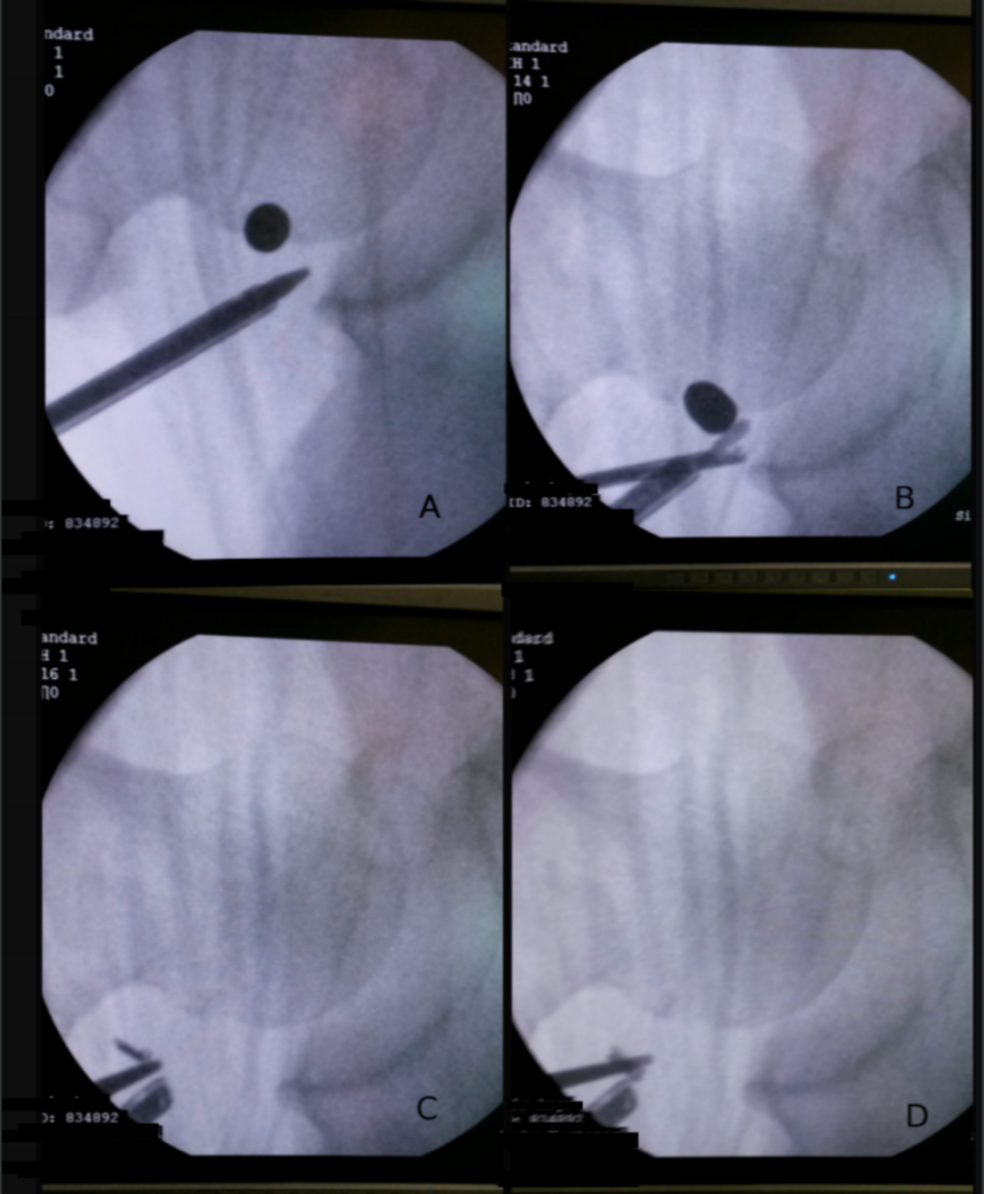
Bullet retrieval attempt and subsequent breaking of the grasper claw A, B: Attempt to retrieve bullet; C, D: Breaking of the grasper claw

Upon establishing sufficient elevation from the femoral head, the grasper was used to grip and, subsequently, pull the bullet out (Figures 2A-2B). After secure grasping of the bullet, suddenly, one of the claws of the grasper broke, releasing the bullet back into the hip joint (Figures 2C-2D). Both the bullet and the claw of the grasper were loose in the patient’s hip joint. No further trials were made by the surgeon to catch them.

**Figure 3:**
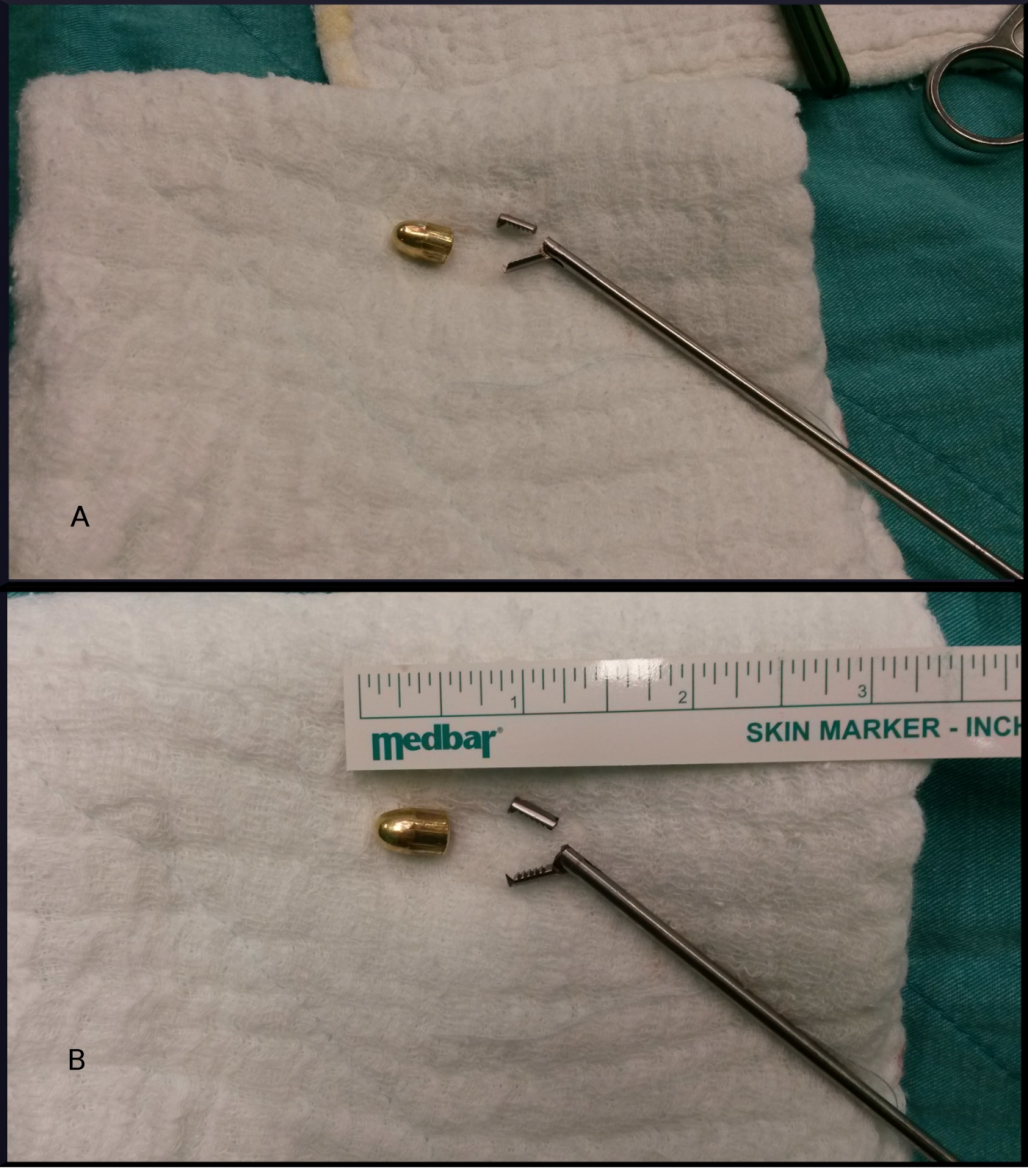
Bullet and broken claw of grasper A. Retrieved bullet and grasper's broken claw, B. Bullet and broken claw with measuring reference

An aggressive bidirectional cut arthroscopic shaver with a diameter of 5.5 mm was utilized as an alternative troubleshooting strategy. The arthroscopic shaver was inserted through the anterior portal with maximum suction pressure in bidirectional mode. The suction pressure of the arthroscopic shaver caused the loose broken claw piece to migrate from the joint space into the mouth of the shaver. The catch was confirmed via arthroscopic view and intraoperative fluoroscopic view. The broken claw of the grasper was sucked in but got stuck at the teeth of the shaver. A long surgical clamp was used to remove the grasper claw from the shaver via the posterolateral portal. Finally, the arthroscopic shaver was introduced, and the suction was deployed again with high pressure to keep the bullet stable at the shaver’s mouth. The bullet was levered out using the long clamp through the posterolateral portal without further complications (Figures 3A-3B).

As a postoperative practice, cephalosporin was administered and physical therapy was scheduled for the next day. The therapy included weight-bearing allowance training as well as balanced gait lessons. The patient was discharged from the hospital with an oral antibiotic under the general surgeon’s prescriptions.

During a two-year follow-up program, no postoperative complications were reported. No evidence of arthritis, neurovascular complications, or infectious symptoms were encountered. Additionally, no ambulation complaints were stated from the patient during the evaluation. The patient was healed and became ambulant.

## Discussion

In the recent past, hip arthroscopy has been relied upon as the best alternative to the conventional open arthrotomy. This is due to its minimal invasive prospect of accessing the hip joint. Past reports described the technique as effective both as a therapeutic and a diagnostic tool for a variety of disorders.

Most reports emphasize hip arthroscopy as a suitable approach in the removal of loose bodies such as bullets. Four reports focused on the removal of a bullet from the hip joint via the acetabulum side or the femoral head [2,5-7]. Of all the published reports, none discussed the removal of two loose bodies from the femoral head of a hip joint.

In this case, we focus on bullet removal from the femoral head of a hip joint, as well as a troubleshooting technique, in a situation where the claw of the grasper breaks inside the joint. This approach proved to be reliable and effective, because the shortcomings of the standard open arthrotomy were avoided, including the need for traction, alternate portals, damage of both acetabular labrum and articular cartilage. Removal of both the bullet and the grasper’s claw tooth is presented as a troubleshooting technique in the worst-case scenario when the claw is broken while being engaged.

A literature review of the arthroscopic loose body removal techniques from the hip joint is shown in Table 1.

**Table 1:** Literature review of the arthroscopic loose body removal techniques from the hip joint

Author	Highlight in the Article
Budeyri A. (2018)	Described the effective removal of a bullet and a grasper's broken claw from the hip joint with the aid of the maximum suction pressure of the arthroscopic shaver.
Guanche, C.A. (2012)	Described the removal of a bullet from the hip joint via the acetabulum.
Singleton, S. B., Joshi, A., Schwartz, M.A., et al. (2005)	Described the arthroscopic removal of a bullet from a patient's hip joint after entering the abdomen and traversing the rectum, before getting lodged in the acetabulum dome.
Mineo, R.C., & Gittins, M.E. (2003)	Described the removal of a bullet from the hip joint via the acetabulum.
Teloken, M.A., Schmietd, I., & Tomlinson, D.P. (2002)	Described the effective removal of a bullet from the right femoral head using the inferomedial arthroscopic technique.
Cory, J.W., & Ruch, D.S. (1998)	Described the first arthroscopic removal of a foreign body such as a bullet.
Gross, R. (1977)	Guanche's technique was used in the routine anterolateral and anterior arthroscopic hip portals performed on the patient in this case study.
Burman, M.S. (1931)	Burman's published work in 1931 shaped the practice of hip arthroscopy.

Singleton et al. (2005) reported the arthroscopic removal of a bullet from the patient’s hip joint after entering the abdomen and traversing the rectum before getting lodged in the acetabulum dome [6].

Teloken et al. (2002) explained the effective removal of a bullet from the right femoral head using the inferomedial arthroscopic technique [7]. A large pituitary rongeur was used to securely grasp both the chondral fragment and the bullet, followed by copious lavage. The author pointed out that the patient remained postoperatively non-weight-bearing for six weeks.

Mineo et al. (2003) reported a case in which hip arthroscopy was chosen to remove a bullet embedded in the acetabulum [5]. The patient sustained a low-velocity gunshot wound. The bullet entered the anterior abdomen, traversed the urinary bladder and inner walls of the acetabulum before being embedded in the intra-articular region of the subchondral bone of the hip joint. Hip arthroscopy was employed in removing the intra-articular debris, examining the chondral surface to prevent damage to the femoral head and late lead intoxication sequelae.

Cory & Ruch (1998) published a report explaining the arthroscopic removal of a .44 caliber bullet from the hip joint [2]. Bullet manipulation involved both the standard anterior portal and anterior lateral portal. A large pituitary rongeur was used to securely grasp and remove the bullet via the anterior portal.

Admitting the recent advances in hip arthroscopy, there are no previous reports describing the removal of two loose bodies - the bullet and the broken grasper’s claw tooth - from the hip joint. Moreover, we are not aware of previous reports explaining a troubleshooting technique in situations where the grasper’s claw gets loose or, even worse, breaks inside the hip joint during bullet grasping. As a rule of thumb, we avoid recommending over-reliance on this specific prospect of hip joint surgical complications.

Furthermore, an effective mechanism to troubleshoot a worst-case scenario, where the grasper gets loose or breaks inside the joint, has been sufficiently introduced. Surgeons should be aware of their instruments’ long-term stress-shielding and the metal fatigue at the claw joints of graspers. In our technical note, we believe that these two are the most major reasons for claw breakage, even under mild grasping forces. The operation may be complicated when there is no sterile substitute grasper or thin and long clamps for hip arthroscopy present. Especially in cases of a deep hip joint, the basic operational instruments may be inadequate for challenging scenarios and their accurate troubleshooting. Graspers with adequate size and durability should be chosen in order to remove the intraarticular foreign bodies effectively. Under certain circumstances, conventional surgical clamps may be useful to securely grasp and remove the intraarticular loose bodies.

Regardless of the number of instruments utilized, the technique is advantageous due to the fact that the operative time was greatly reduced. Moreover, iatrogenic trauma associated with the standard arthrotomy technique was prevented, including damage to the acetabular labrum and articular cartilage. Finally, our approach provides an effective troubleshooting technique in circumstances when the grasper loosens or breaks inside the hip joint during the surgical operation. Future case reports to come will assess both the risks and benefits of this new technique.

## Conclusions

Hip arthroscopy is, without any doubt, a reliable technique with great benefits for the removal of foreign bodies from major joints. We have successfully presented an unusual case with two loose bodies in the hip joint and an interesting approach for their surgical removal. Our technique highlights the use of the surgical shaver suction for stabilizing loose objects in the hip joint. Regarding the conventional arthrotomy, we point to the minimal surgical complications, the shortened rehabilitation, and the reduced postoperative discomfort.
